# A Novel Method for Antibiotic Detection in Milk Based on Competitive Magnetic Immunodetection

**DOI:** 10.3390/foods9121773

**Published:** 2020-11-30

**Authors:** Jan Pietschmann, Dominik Dittmann, Holger Spiegel, Hans-Joachim Krause, Florian Schröper

**Affiliations:** 1 Fraunhofer Institute for Molecular Biology and Applied Ecology IME, Forckenbeckstraße 6, 52074 Aachen, Germany; dominik.dittmann@ime.fraunhofer.de (D.D.); holger.spiegel@ime.fraunhofer.de (H.S.); florian.schroeper@ime.fraunhofer.de (F.S.); 2 Institute of Biological Information Processing, Bioelectronics IBI-3, Forschungszentrum Jülich, 52428 Jülich, Germany; h.-j.krause@fz-juelich.de

**Keywords:** frequency mixing technology, immunofiltration, magnetic beads

## Abstract

The misuse of antibiotics as well as incorrect dosage or insufficient time for detoxification can result in the presence of pharmacologically active molecules in fresh milk. Hence, in many countries, commercially available milk has to be tested with immunological, chromatographic or microbiological analytical methods to avoid consumption of antibiotic residues. Here a novel, sensitive and portable assay setup for the detection and quantification of penicillin and kanamycin in whole fat milk (WFM) based on competitive magnetic immunodetection (cMID) is described and assay accuracy determined. For this, penicillin G and kanamycin-conjugates were generated and coated onto a matrix of immunofiltration columns (IFC). Biotinylated penicillin G or kanamycin-specific antibodies were pre-incubated with antibiotics-containing samples and subsequently applied onto IFC to determine the concentration of antibiotics through the competition of antibody-binding to the antibiotic-conjugate molecules. Bound antibodies were labeled with streptavidin-coated magnetic particles and quantified using frequency magnetic mixing technology. Based on calibration measurements in WFM with detection limits of 1.33 ng·mL^−1^ for penicillin G and 1.0 ng·mL^−1^ for kanamycin, spiked WFM samples were analyzed, revealing highly accurate recovery rates and assay precision. Our results demonstrate the suitability of cMID-based competition assay for reliable and easy on-site testing of milk.

## 1. Introduction

**Figure 1:**
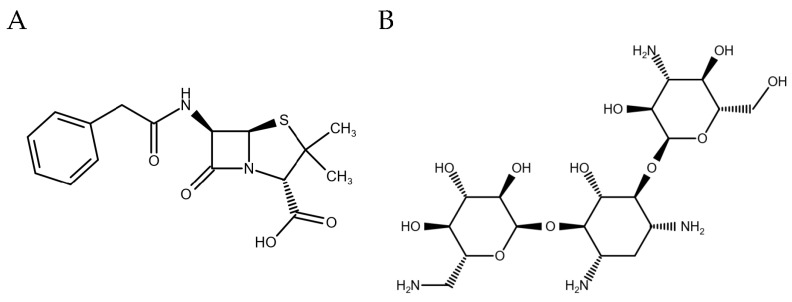
Chemical structures of (**A**) penicillin and (**B**) kanamycin.

Antibiotics are small molecules either produced by different molds such *Penicillium* species or bacteria such as *Streptomyces* spp., or artificially synthesized, and can inhibit the growth of various pathogens [1,2]. Worldwide, annually more than 60,000 tons of antibiotics are used to treat bacterial infectious diseases in animal husbandry with numbers being expected to reach more than 100,000 tons by the year 2030 [3]. Antibiotics belonging to the classes of β-lactams such as penicillin (Figure 1A) and aminoglycosides such as kanamycin (Figure 1B) are primarily used for treatment of infectious diseases [4,5,6,7,8]. Different studies showed that a large part of the antibiotics used in animal husbandry can be released undegraded, with antimicrobial activity, into the environment [9]. Consumption of contaminated food leads to repeated and, consequently, long exposure times to these antibiotics, which poses a major threat for public health. The risks include development of bacterial resistances, allergies and hypersensitive reactions [3,6,8,10,11]. Mostly, antibiotic residues found in food samples are caused by injudicious usage, such as use as growth promotors, incorrect dosage or not maintaining proper detoxification times, e.g., affected by a lack of proper farmer education or awareness [7,12,13].

Due to the growing risk of overexposure to antibiotics in animal derived foods such as meat, milk or eggs, countries of the European Union (EU) have defined residue limits which specify the acceptable dosage of an antibiotic that will probably not affect consumer health. For the EU, these maximum residue limits (MRL) are defined in the European Union Commission Regulation No. 37/2010 and are set at 4 ng·mL^−1^ for benzylpenicillin (penicillin G) and 150 ng·mL^−1^ for kanamycin in milk, which are comparable to those set in the US [14].

Currently used detection methods are mostly based on chromatographic, immunological and microbiological test procedures [7,15,16,17]. Especially in the field of chromatographic methods, LC-MS/MS-based analytical technologies enable a highly sensitive and simultaneous detection of multiple antibiotic residues within a single sample [18,19,20]. Using chromatographic methods, multiresidue analytics with detection limits lower than 1 ng·mL^−1^ for many antibiotics can be performed [21]. However, this method is restricted to analytical laboratories due to the need for highly trained staff and cost-intensive laboratory-based equipment [15,16,22]. Nowadays, immunological tests such as ELISA or lateral flow assays (LFAs) as well as microbial test kits are commonly used for monitoring of milk samples [7,15,17]. LFAs are simple, easy-to-handle and are usually performed within minutes, which makes them easy to use, even for untrained personnel [22]. However, such LFAs lack sensitivity and a quantitative measurement is typically not possible. In contrast, ELISAs have a high sensitivity and are at least semi-quantitative, but their dynamic range of detection is quite low and additionally they lack speed due to long incubation times [23]. Furthermore, experienced staff are needed for performing these assays. Microbiological tests such as the Brilliant Black Reduction Test are easy in procedure but also need laboratory-based equipment. By the application of a (probably) antibiotic-containing sample onto a reference microorganism, bacteria growth is inhibited and a colorimetric change cannot be seen. Although it is a quite simple procedure, it needs a few hours to enable bacteria growth and it lacks specificity since bacterial growth is inhibited by all kinds of antibiotics. Additionally, by analyzing low-contaminated samples, visual interpretation could be difficult, which increases the rate of false negative results [4,24,25]. However, if the milk samples seem to be above the MRL and the previously described tests show positive results, the suspect milk must be sent to analytical laboratories where it is retested for confirmation in accordance with regulatory requirements. Here, a quantitative detection of antibiotic residues is done, mainly using LC-MS/MS-based analytical methods. With those cost-intensive, quantitative results, farmers can calculate when the milk from treated husbandries has to be discarded.

In previous studies, magnetic immunodetection (MID) has been successfully employed for detection and quantification of human as well as plant pathogens and such bacterial toxins as cholera toxin B by sensing superparamagnetic particles [26,27,28,29]. In a recent study, a highly sensitive and quantitative detection of aflatoxin B1 has been demonstrated based on newly developed competitive magnetic immunodetection (cMID) [23]. Using frequency mixing magnetic detection (FMMD), a dose-depending measuring signal is obtained in a portable handheld measuring FMMD device [23,26,29,30]. A detailed explanation of FMMD is given in [23,29,31]. In a competitive MID setup, the obtained measuring signal is reciprocally correlated with the amount of analyte in the sample. Easy sample handling and, especially, the possibility for battery-driven operation of the handheld FMMD device, enables on-site analytics and readout without need for special laboratory equipment or electrical infrastructure. Due to several drawbacks of the currently used immunological or microbiological detection technologies and their mainly qualitative results with limited range of quantification, we developed a highly sensitive assay based on cMID for efficient detection, in combination with quantification of penicillin G and kanamycin, in milk samples. For this, penicillin-bovine serum albumin (BSA) and kanamycin-BSA conjugates were synthesized and affinity of monoclonal antibodies (mAb) were determined by dose-response analysis. Afterward, cMID assays were established and highly specific assay accuracy and quantification was demonstrated by spiking different concentrations of either penicillin G or kanamycin in whole fat milk (WFM) and evaluating recovery rates. With this demonstrated proof-of-concept assay, farmers can control their milk directly on-site and can estimate the needed detoxification time without cost-intensive analytics in regulatory laboratories.

## 2. Materials and Methods

### 2.1. Materials and Chemicals

Dimethyl sulfoxide (DMSO), 1-Ethyl-3-(3-dimethylaminopropyl)carbodiimide hydrochloride, EZ-Link™ NHS-PEG4 Biotinylation Kit, and NHS were acquired from Merck KGaA, Darmstadt, Germany. Albumin Fraction V (biotin-free), KCl, KH_2_PO_4_, NaCl, Na_2_(CO_3_), NaHCO_3_, Na_2_HPO_4_ × 12 H_2_O and Tween-20 were purchased from Carl Roth, Karlsruhe, Germany. Immuno-filtration columns (ABICAP HP columns) were purchased from Senova Gesellschaft für Biowissenschaft und Technik mbH, Weimar, Germany. Anti-penicillin G monoclonal antibody was kindly provided by the Milchprüfring Bayern e.V. (MPR), Wolnzach, Germany. Anti-kanamycin monoclonal antibody (article number CSB-MA000511I0m) was purchased from Cusabio, Wuhan, China. Detection antibody goat anti-mouse IgG coupled to HRPO (article number 115-035-008) was purchased from Jackson ImmunoResearch Europe Ltd., Ely, UK. Penicillin G and kanamycin A were acquired from Duchefa Biochemie, Haarlem, Netherlands. Magnetic particles with streptavidin-functionalized shell and a hydrodynamic diameter of 70 nm [synomag^®^-D, article number 104-19-701] were purchased from micromod Partikeltechnologie GmbH, Rostock, Germany.

Coupling buffer, phosphate buffered saline (PBS), PBS-Tween (PBS-T) as well as blocking solutions for ELISA and magnetic immunodetection were prepared as described in [23].

### 2.2. Generation of Antibiotic-BSA Conjugates

#### 2.2.1. Penicillin-BSA

Penicillin-BSA conjugate was prepared according to an adapted protocol described by Venkataramana and colleagues (2015) [32]. For this, 5.6 mg NHS (43 µmol) and 8 mg EDC-HCl (41 µmol) were dissolved in 500 µL DMSO. The solution was then transferred to 5 mg penicillin G (15 µmol) and incubated for two hours at room temperature in dark surroundings, followed by an overnight incubation at 4 °C in dark surroundings. On the next day, the solution was added dropwise to 10 mg of BSA (0.15 µmol). Afterward, 2 mL of carbonate buffer (pH 9.6) was added to the solution, followed by an incubation for 2 h at room temperature (RT) in dark surroundings. The solution was then dialyzed for four days against daily exchanged 5 l PBS (pH 7.4). After sterile filtration through a filter with a pore diameter of 0.22 µm, the solution was stored at 4 °C until usage.

#### 2.2.2. Kanamycin-BSA

Kanamycin-BSA conjugate was prepared according to a protocol described by Haasnoot et al. (1999) [33]. Firstly, 34 mg kanamycin A (701 µmol) and 10 mg BSA (0.15 µmol) were dissolved in 1 mL MilliQ-water. Afterward, 383 mg EDC-HCl (2 mmol) were dissolved in 1 mL MilliQ-water. This solution was added dropwise to the kanamycin and BSA solution and incubated for 2 h at RT while shaking. The solution was then dialyzed against daily exchanged 5 l PBS (pH 7.4) for four days at 4 °C in dark surroundings. After sterile filtration through a filter with a pore diameter of 0.22 µm, the solution was stored at 4 °C until usage.

### 2.3. Determination of Protein Concentration

Protein concentration of each antibiotic-BSA conjugate was determined after final sterile filtration with Bradford protein assay using the Roti^®^-Quant Bradford reagent (Carl Roth) against BSA as reference. The assay was performed according to the manufacturer’s instructions. Briefly, a serial dilution of samples was prepared in a 96-well plate and 200 µL of Bradford reagent was added. After an incubation of 5 min at room temperature, the absorbance was measured at a wavelength of 595 nm.

### 2.4. Determination of Antibody Affinity

Affinity of the monoclonal antibodies to the antibiotic-BSA conjugates was determined with an ELISA. All incubation steps were performed for one hour at RT in dark surroundings. After each step, plates were washed by rinsing the wells three times with 200 µL PBS-T. A high-binding 96-well microtiter plate (Greiner Bio-One) was coated with 100 µL per well of 2 µg/mL penicillin-BSA or kanamycin-BSA. Afterward, remaining free binding sites were blocked with 200 µL per well of 5% (*w*/*v*) skimmed milk.

A serial dilution of anti-penicillin G or anti-kanamycin monoclonal antibodies with concentrations ranging from 9.76 ng·mL^−1^ up to 1250 ng·mL^−1^ was prepared and applied onto coated and blocked microtiter plate. Following incubation and washing, 100 µL of 80 ng·mL^−1^ detection antibody, diluted in PBS, was added to each well and incubated. Following a final washing step, 100 µL of 1 mg·mL^−1^ ABTS substrate in ABTS buffer was applied and absorption was measured at 405 nm wavelength after 10 min of incubation.

### 2.5. Preparation of Immunofiltration Columns

The equilibration of immunofiltration columns (IFCs) was performed as previously described [23,29]. For degassing of IFCs, the columns were placed in ethanol (96%) inside of a desiccator at a pressure of—0.8 bar for 20 min. Afterward, the columns were washed with each 750 µL ethanol-water (50/50), MilliQ-water and twice with carbonate buffer (pH 9.6). Matrices were coated by rinsing 500 µL of antibiotic-BSA conjugate solution (3.5 µg·mL^−1^), diluted in coupling buffer, through each column. After an incubation of one hour at RT, columns were washed by rinsing 750 µL PBS through the matrix. Subsequently, remaining free binding sites inside the matrix were blocked by adding twice 750 µL of a 1% (*w*/*v*) PBS-BSA solution onto each column. After second application an incubation of 60 min was performed. After washing by applying twice 750 µL of PBS onto columns, the assay can be performed, or the columns can be stored at 4 °C in PBS for 14 days.

### 2.6. cMID Calibration Curve Analysis

For preparation of cMID calibration curve analysis, a pre-incubation of free antibiotics and biotinylated antibody was performed. Biotinylation of antibody was performed as described in [23]. For this, serially diluted penicillin G or kanamycin samples in PBS, with concentrations ranging from 0.011 ng·mL^−1^ to 3000 ng·mL^−1^ for penicillin G and 0.0057 ng·mL^−1^ to 1500 ng·mL^−1^ for kanamycin, were incubated with 1.2 µg·mL^−1^ biotinylated antibody, also diluted in PBS. As positive control, a sample of respective biotinylated antibody without the addition of antibiotic was prepared for determining the highest possible measuring signal, later called B0 signal. After incubating the sample for one hour at RT, 500 µL sample volume was applied on coated and blocked columns and also incubated for one hour at room temperature. Subsequently, columns were washed with 750 µL PBS and, afterward, 500 µL of 60 µg·mL^−1^ magnetic particles were rinsed through the column and incubated for one hour at room temperature. After final washing with 750 µL PBS through the matrix, the columns were measured using a portable FMMD magnetic reader.

### 2.7. Frequency Mixing Magnetic Detection (FMMD)

The magnetic nanoparticle markers were detected using a custom-made magnetic reader consisting of a measurement head with excitation and detection coils and an electronic readout [29]. In brief, the sample containing the magnetic particles is exposed to a magnetic field consisting of two distinct frequencies, a high frequency field of approximately a milli-tesla at *f*_1_ = 49 kHz and a low-frequency field of about ten milli-tesla at *f*_2_ = 61 Hz. Due to the particles’ nonlinear superparamagnetic magnetization, intermodulation products are generated and picked up in the detection coil. The dominant mixing component at frequency *f*_1_ + 2·*f*_2_ is demodulated. Its amplitude is proportional to the particle concentration in the sample. Details of the measurement principle and of the setup are given in [23,28,29].

### 2.8. Sample Preparation and cMID in Milk

The calibration curve in milk was prepared as described above. For this purpose, unconjugated antibiotics were dissolved in whole milk (3.5% total fat) instead of PBS. For determination of assay accuracy, spiked milk samples were prepared in whole milk (3.5% total fat) at concentrations of 4 ng·mL^−1^, 8 ng·mL^−1^, 20 ng·mL^−1^, 40 ng·mL^−1^ and 200 ng·mL^−1^ for penicillin G and 1 ng·mL^−1^, 10 ng·mL^−1^ and 50 ng·mL^−1^ for kanamycin. The spiked samples were incubated with 1.2 µg·mL^−1^ of biotinylated antibody diluted in PBS. After application of samples onto pre-coated and blocked IFCs, the assay procedure was carried out as described above.

### 2.9. Data Analysis

For all data analysis and data fitting using Hill Fit, GraphPad Prism 8.3.1 and Hill parameters calculated by GraphPad Prism were used. To compare sample intensities in relation to highest maximum signal (sample without competitor), Equation (1) was used. Calculating limit of detection (LOD) or maximum of detection (MOD) on the signal scale, Equation (2) or (3), respectively, were used. For calculation of concentration of LOD, MOD as well as spiked samples, Equation (4) was used. Recovery rates of spiked samples were determined using Equation (5):

(1) $$B/\mathrm{BO}\;\mathrm{ratio}=\frac{Measuring\;signal_{Sample}}{Average\;measuring\;signal_{Sample\;without\;antibiotic}}$$

(2) $${\mathrm{Signal}}_{\mathrm{Limit}\;\mathrm{of}\;\mathrm{Detection}}=Average\;B/B0\;signal_{Saturated\;samples}-3\times SD_{Saturated\;samples}$$

(3) $${\mathrm{Signal}}_{\mathrm{Maximum}\;\mathrm{of}\;\mathrm{Detection}}=Average\;B/B0\;signal_{Background\;samples}+3\times SD_{Background\;samples}$$

(4) $${\mathrm{Concentration}}_{\mathrm{Sample}}={\left[\frac{IC_{50}^{h}\times \left(B/B0\;Signal_{Sample}-B_{max}\right)\;}{B/B0\;Signal_{Sample}^{Hill\;Slope}}\right]}^{-\frac{1}{Hill\;Slope}}$$

(5) $$\mathrm{Recovery}\;\mathrm{Rate}=\frac{Concentration\;detected}{Concentration\;spiked}\times 100\;\left[\%\right]\;$$

## 3. Results and Discussion

### 3.1. Generation of Penicillin-BSA and Kanamycin-BSA Conjugates and Antibody Affinity Determination

**Figure 2:**
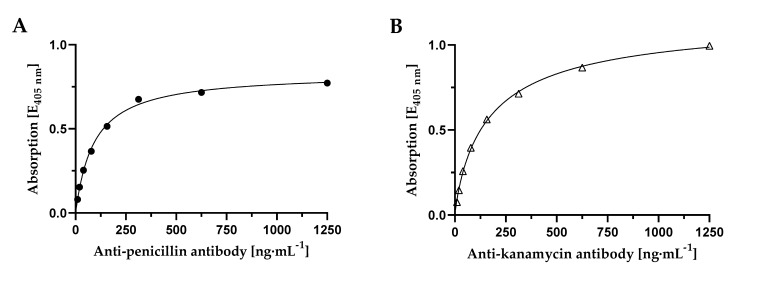
Affinity determination of (**A**) anti-penicillin G specific mAb against penicillin G-BSA conjugate and (**B**) anti-kanamycin specific mAb against kanamycin-BSA conjugate. For both, a microtiter plate was coated with respective antibiotic-BSA conjugate and remaining binding sites were blocked. Subsequently, antibody dilutions in the range from 9.76 ng·mL^−1^ to 1250 ng·mL^−1^ were applied and afterward labelled with mouse IgG-specific HRPO-conjugated secondary antibody. Absorbance was measured after 10 min of ABTS substrate incubation. *n* = 1.

In order to establish a reliable assay for highly sensitive detection and quantification of penicillin and kanamycin in milk samples, penicillin-BSA and kanamycin-BSA conjugates had to be prepared and tested regarding their binding capacity with respective monoclonal antibodies (mAb). For testing the affinity of corresponding mAb towards self-conjugated penicillin-BSA conjugate (Figure 2A) or kanamycin-BSA conjugate (Figure 2B) in an ELISA, antibodies were titrated ranging from 9.76 ng·mL^−1^ up to 1250 ng·mL^−1^ against coated antibiotic-conjugate. A high affinity of respective antibody against their antigen-conjugate could be detected with EC_50_-values of 91.8 ng·mL^−1^ for penicillin-specific mAb and 177.7 ng·mL^−1^ for kanamycin-specific mAb. Although anti-penicillin antibody resulted in a lower EC_50_-value, a higher kanamycin density on BSA compared to the density of penicillin on BSA could be concluded based on the higher absorbance of anti-kanamycin antibody dose response, achieved after 10 min of substrate incubation. Additionally, a later saturation of measuring signal could be detected with kanamycin-BSA and respective monoclonal antibody, which underlines the higher antigen-density on the carrier protein. However, on those high-affine dose responses, a successful conjugation of penicillin as well as kanamycin with BSA could be demonstrated.

### 3.2. Development of cMID for Detection of Penicllin and Kanamycin in Buffer

**Figure 3:**
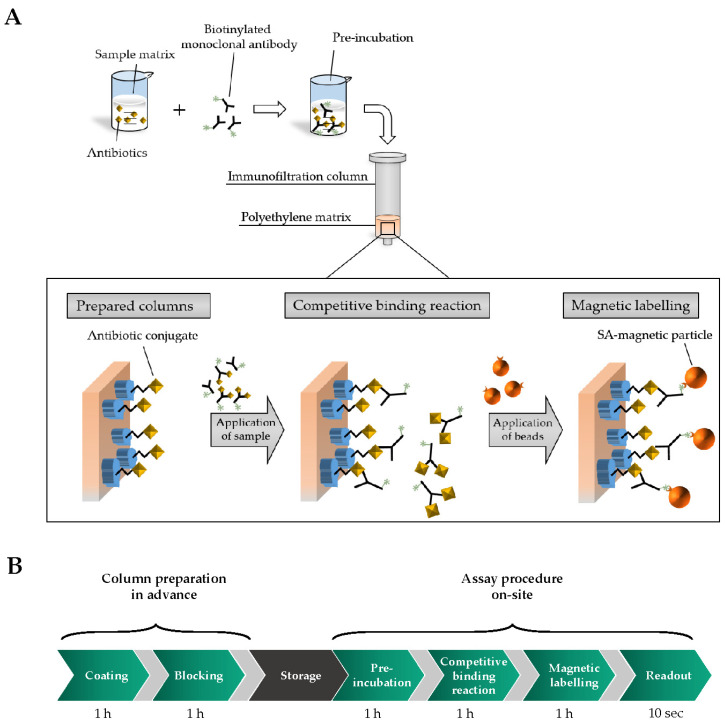
Schematic overview of competitive magnetic immunodetection assay procedure for detection of antibiotics in milk. (**A**) Biotinylated monoclonal antibodies are applied into the sample (either sample buffer or whole fat milk (WFM). Throughout pre-incubation, mAbs bind soluble antibiotic molecules. Afterward, the sample is applied onto antibiotic-conjugate coated and blocked polyethylene matrix of immunofiltration columns (IFCs). Here, a competitive binding reaction of monoclonal antibodies between coated antibiotic-conjugate and soluble antigen results in a retention of non-saturated antibodies within the IFC matrix. Subsequently, retained mAbs are magnetically labeled with streptavidin-functionalized magnetic particles (SA-magnetic particle). Finally, retained SA-magnetic particles can be detected and quantified by means of frequency mixing magnetic detection (FMMD). (**B**) Overview about assay steps with incubation times. Light grey arrows represent washing steps with 2 min of duration.

After successful generation of antibiotic-conjugates and the confirmation of high affinity binding of mAbs, first cMID experiments were performed. For this, biotinylated monoclonal antibodies are pre-incubated with antibiotics-containing samples. Afterward, the mixture is applied onto immunofiltration columns (IFC) containing antibiotic-carrier protein conjugate coated polyethylene matrices. While the sample is flushed through the IFC by gravity flow, a competitive binding reaction of biotinylated monoclonal antibodies between free soluble antigens in the sample and coated antigen-conjugates at the matrix takes place. Hence, non-saturated biotinylated antibodies are retained and subsequently can be magnetically labelled with streptavidin-functionalized superparamagnetic particles based on highly affine streptavidin-biotin reaction. Especially due to the comparable molecular weight and chemical properties of antibiotics and mycotoxins, coating concentration as well as antibody and magnetic particle concentration was adapted from intensive preliminary work of mycotoxin cMID assay development [23]. Additionally, each assay step was set to one hour, enabling full equilibrium of binding reactions. A schematic overview of cMID procedure is demonstrated in Figure 3A and assay times are presented in Figure 3B.

**Figure 4:**
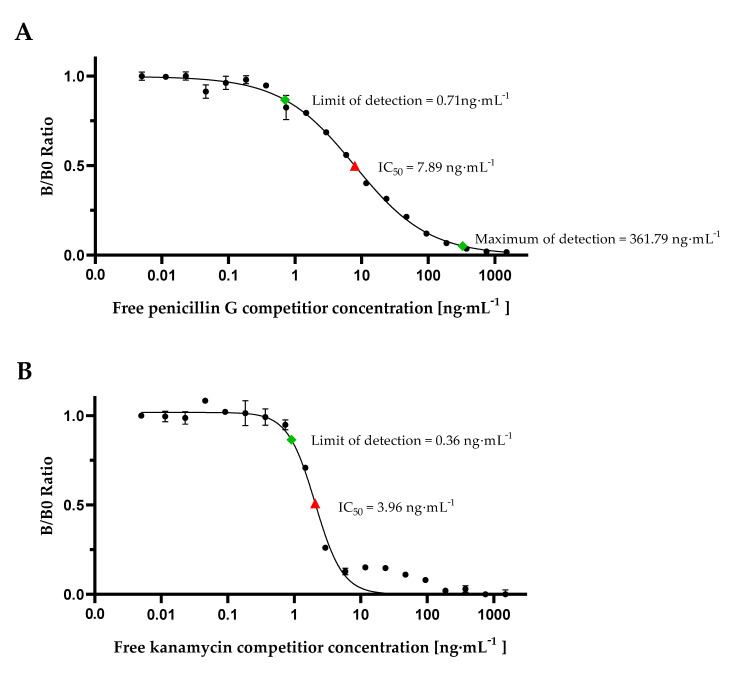
cMID calibration curves for detection of antibiotics in PBS sample buffer. cMID calibration curves for detection of antibiotics in PBS sample buffer. (**A**) Penicillin cMID calibration curve and (**B**) kanamycin cMID calibration curve. For both, columns were coated with respective antibiotic-BSA conjugate and subsequently blocked. After application of each antibiotic diluted from 0.011 ng·mL^−1^ up to 1500 ng·mL^−1^ pre-incubated with 1.2 µg·mL^−1^ of respective mAb, 60 µg·mL^−1^ streptavidin-functionalized superparamagnetic particles were applied and rinsed through the column. Readout was done using frequency mixing magnetic detection (FMMD). Each data point represents mean ± SD (*n* = 2). Limit of detection and, if possible, maximum of detection are indicated by green square. IC_50_ (half maximal inhibitory concentration) is symbolized by red triangle.

Calibration curve experiments with cMID for detection of penicillin G or kanamycin, respectively, were done by diluting penicillin G (Figure 4A) or kanamycin (Figure 4B) in range from 0.011 ng·mL^−1^ up to 1500 ng·mL^−1^ in phosphate-buffered saline (PBS), pre-incubation with respective monoclonal antibodies followed by application on pre-coated IFCs and subsequent magnetic labelling. Measuring signals were recorded using FMMD and normalized by calculating the B/B0 ratios using Equation (1). Calibration results are shown in Figure 4, LOD and MOD values were calculated using Equations (2) and (3) and are indicated.

In our B0 measuring, maximum signals of both calibration curves were at approximately 500 mV. Based on Equation (4), a limit of detection of 0.71 ng·mL^−1^ for penicillin G and a LOD of 0.36 ng·mL^−1^ for kanamycin could be determined. An approximately 2-fold lower IC_50_-value for kanamycin with 3.96 ng·mL^−1^ in comparison to penicillin with 7.89 ng·mL^−1^ demonstrates a higher assay sensitivity. While for penicillin G a MOD of 361.79 ng·mL^−1^ was calculated according to Equation (3), this was not possible for kanamycin due to increasing B/B0 ratio of measuring signals in the range of 11.7 ng·mL^−1^ up to 93.7 ng·mL^−1^. However, for penicillin G it could be demonstrated that the dynamic range (0.71 ng·mL^−1^ up to 361.79 ng·mL^−1^) is greatly increased in comparison to commercially available test kits as EuroProxima Penicillin ELISA (r-biopharm, Darmstadt, Germany). For this ELISA-based assay, a calibration curve ranging from 0.125 ng·mL^−1^ up to 4 ng·mL^−1^ needs to be prepared which just covers the range of MRL for penicillin G. Hence, our results demonstrate an, in general, improved detection of penicillin G and kanamycin by employing our newly developed competitive magnetic immunodetection.

### 3.3. cMID Calibration Measurements in Whole Fat Milk (WFM)

**Figure 5:**
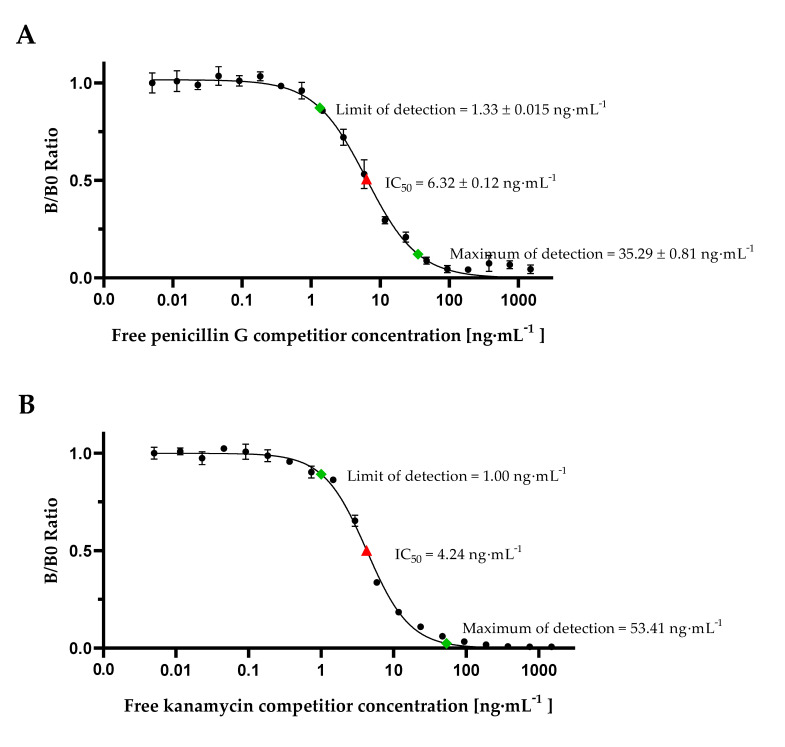
cMID calibration curves for detection of antibiotics spiked in whole fat milk. (**A**) Penicillin G calibration curve and (**B**) kanamycin calibration curve. Both antibiotics were diluted from 0.011 ng·mL^−1^ up to 1500 ng·mL^−1^ in whole fat milk and were pre-incubated with 1.2 µg·mL^−1^ of respective biotinylated monoclonal antibodies (mAb). Afterward, the samples were applied onto 3.5 µg·mL^−1^ antibiotic-BSA conjugate coated and blocked IFC. Finally, 60 µg·mL^−1^ superparamagnetic particles functionalized with streptavidin were applied and rinsed through the column. Readout was done using FMMD. Each data point represents mean ± SD. For (**A**) penicillin, data points were averaged from two independent calibration curve experiments (*n* = 4) and for (**B**) kanamycin each data point represents the mean ± SD of *n* = 2. Limit of detection and maximum of detection are indicated by green square. IC_50_ (half maximal inhibitory concentration) is symbolized by red triangle.

With the previously shown calibration curve analysis in PBS, an efficient detection of antibiotics could be demonstrated. However, for performing the assay in WFM, studies of matrix interference, which could be reasoned by the inhibitory effects of fatty acids on antibody-binding or hydrophobic interaction of antigen, had to be done. To avoid such matrix effects, samples can be diluted in assay buffer, e.g., PBS, resulting in strong dilution of interfering substances. However, diluting the samples to be analyzed would also decrease the assay accuracy. Especially in the case of penicillin G with a low MRL of just 4 ng·mL^−1^, too high dilution would increase the possibility of false negative results because penicillin G concentration might become lower than the detection limit of 0.71 ng·mL^−1^ in PBS. Hence, in this study, calibration curves were prepared in commercially available, controlled whole fat milk instead of PBS to analyze the influence of milk matrix effects on sensitivity and to determine cMID assay applicability (Figure 5). For this purpose, penicillin G (Figure 5A) and kanamycin (Figure 5B) were diluted in WFM in the same ranges as previously in PBS (Figure 4). For cMID analysis the samples were diluted twofold in PBS buffer, due to the addition of the respective antibody.

Especially in the case of penicillin G detection, an influence of WFM as matrix could be observed by the three-fold reduction of B0 measuring signal to a maximum of approximately 150 mV. Furthermore, an approximately ten-fold reduced dynamic detection range from 1.33 ± 0.015 ng·mL^−1^ up to 35.29 ± 0.81 ng·mL^−1^ compared to measurements in PBS was obtained. The reduced B0 signal as well as the reduced dynamic range of detection could be attributed to a lipid-mediated interference, which could inhibit the binding of monoclonal antibodies to coated antigen [34]. Compared to commercial ELISA kits such as EuroProxima Penicillin ELISA (r-biopharm, Darmstadt, Germany), in our approach a higher LOD of 1.33 ± 0.015 ng·mL^−1^ compared to 0.08 ng·mL^−1^ was obtained. However, the dynamic range obtained was still approximately ten-fold higher than in ELISA (up to 35.29 ± 0.81 ng·mL^−1^). Currently further optimization experiments are planned to improve coating and antibody concentrations in a similar way as performed during our mycotoxin cMID assay development [23]. Hence, a further enhancement of assay sensitivity can be expected. Interestingly, in the case of IC_50_-value, no relevant negative matrix interference in WFM could be detected for cMID. In contrast to penicillin G, for kanamycin cMID no reduction of the B0 signal was observed. A slight reduction of dynamic detection range from 1.00 ng·mL^−1^ up to 53.41 ng·mL^−1^ was determined, suggesting only a weak matrix effect. The IC_50_-value of 4.24 ng·mL^−1^ was also comparable to the value from PBS calibration experiments. The obtained assay parameters were comparable to laboratory-based ELISA kits as e.g., Kanamycin ELISA kit (Creative Diagnostics, New York, USA) with a detection range from 0.5 ng·mL^−1^ up to 40.5 ng·mL^−1^.

### 3.4. Spiked Sample Analysis and Determination of Recovery Rate

For determination of assay accuracy, milk samples were spiked with different concentrations of respective antibiotics and recovery rates were calculated using the fit function of previously obtained calibration curves (Figure 5). Penicillin was spiked at industrially relevant concentrations of 2 ng·mL^−1^, 4 ng·mL^−1^, 10 ng·mL^−1^ and 20 ng·mL^−1^, and kanamycin at concentrations of 1 ng·mL^−1^, 10 ng·mL^−1^ and 50 ng·mL^−1^ into milk samples. For penicillin G, recovery rates between 91.4% ± 14.9% up to 112.5% ± 13.1% could be achieved (Table 1). Compared to commercially available kits with maximally achieved recoveries of 86 ± 7% in milk (EuroProxima Penicillin ELISA, r-biopharm, Darmstadt, Germany), our newly developed assay demonstrated a superior assay performance. For kanamycin, recovery rates of 87.8% ± 4.3% for 1 ng·mL^−1^ and 94.1% ± 0.1% for 4 ng·mL^−1^ spiked samples were obtained (Table 1), which also demonstrate a higher assay accuracy compared to commercially available Kanamycin ELISA kit (Creative Diagnostics, New York, NY, USA) with an accuracy of 85% ± 10%. For the last spiked kanamycin sample with a concentration of 50 ng·mL^−1^, a recovery rate of only 52.5% ± 0.7% was achieved. However, this result is not surprising since with this concentration the maximum detection limit of 53.41 ng·mL^−1^ is almost reached, making a reliable quantification difficult. Hence, these results reveal a high assay accuracy, a high sensitivity, and a good applicability of developed cMID for detecting antibiotics in milk samples.

Hence, our results reveal a high assay accuracy, a high sensitivity and a good applicability of developed cMID for detecting and quantifying antibiotics in milk samples. Thus, the potential as a highly accurate detection and quantification method was demonstrated although just a limited number of spiked WFM samples were used. With this, a proof-of-concept approach was demonstrated. Before translating the method to commercial application, an extensive as well as a fit-for-purpose (FFP) validation process should be carried out to further confirm accuracy, reproducibility and robustness. A further applicability of this assay format could be a simpler qualitative detection of antibiotic residues in milk samples. In such a screening setup, the measured magnetic signal resulting from a contamination as low as the MRL could be defined as threshold. By this, a resulting measuring signal either defines a sample as contaminated above or below the MRL. To demonstrate this specific applicability, an additional FFP validation should be performed. Practically, additional spiking experiments with at least 20 different blank samples as well as 20 spiked samples containing relevant concentrations of respective antibiotics, at approximately 0.5 times the MRL, should be performed. According to this, for penicillin G a concentration of 2 ng·mL^−1^ should be used. In case of kanamycin an even lower concentrations of 10 ng·mL^−1^ would be recommended due to the higher assay accuracy of 94.1% ± 0.1% (Table 1). Such additional validation experiments would then be suitable to further confirm the practicability and overall assay accuracy of our method.

## 4. Conclusions

We demonstrate a newly developed proof-of-concept method for detection of penicillin G and kanamycin in milk. With our cMID approach, a detection of penicillin G in the range from 1.33 ± 0.015 ng·mL^−1^ up to 35.29 ± 0.81 ng·mL^−1^ in WFM with an accuracy ranging from 91.4% up to 112.5% and detection of kanamycin contaminations in WFM ranging from 1.00 ng·mL^−1^ up to 53.41 ng·mL^−1,^ with recovery rates between 87.8% up to 94.1% in the linear range of our calibration curve, is possible. Based on the MRL of 4 ng·mL^−1^ for penicillin, a direct analysis of milk can be performed following the addition of antibody, since no further preparation steps of milk are necessary. For kanamycin, samples should be at least diluted threefold, although no matrix effects could be noticed. This is reasoned by the high assay sensitivity. Milk samples with kanamycin concentrations lower than the MRL will be detected as positive, resulting in false positive assay outcome. However, compared to commercially ELISA kits, a higher range of detection of penicillin G and kanamycin was demonstrated. By spiking whole fat milk samples with different concentrations of these antibiotics and re-calculating concentrations using corresponding calibration measurements, a superior assay accuracy ranging from 91.4% up to 112.5% for penicillin G detection and 87.8% up to 94.1% in the linear range of calibration curve for kanamycin detection could furthermore be demonstrated.

Our easily applicable assay setup in combination with the handheld FMMD readout device could, in contrast to other laboratory based standard procedures, be suited for fast on-site testing and thus be applicable even for farmers. This easy assay setup with broad range of quantification might then lead to better estimation of necessary detoxification time after antibiotic treatment of milk cows. However, when using raw milk, an even stronger matrix interference as described in Section 3.3 when analyzing penicillin G contaminations could be expected, which further diminishes the dynamic range of detection. For this, further preparation steps, such as the usage of a detergent, should be tested, reducing the risk of lipid-mediated matrix interference by disrupting lipid interaction. Nevertheless, if a too strong reduction of dynamic range of detection is observed, the assay could still be used as qualitative screening approach after further, appropriate validation, as suggested in Section 3.4.

However, for fast on-site testing the overall assay time needs to be further reduced. Hence, in further studies a reduction of assay steps and time will be addressed by, e.g., testing optimized incubation times for pre-incubation, competitive binding reaction and magnetic labelling. Based on our experience, this should result in an approximately six-fold reduced assay time from 3 h (Figure 3B) to less than 30 min, as demonstrated for a sandwich based MID approach by Rettcher et al. (2015) [29]. Additionally, the assay setup can be easily adapted for detecting other antibiotics in milk, just by exchanging the coating antigen and using other specific antibodies. Our approach might thus be a platform-like assay system for antibiotic detection in general. By the distinction of different magnetic particle types by their characteristic measuring signals, as demonstrated by Achtsnicht et al. (2019) [35], even a multiplex detection of several antibiotics in one sample is feasible, which will be addressed in further studies. Hence, by pre-functionalizing different magnetic particles with monoclonal antibodies targeting several antibiotics, multiple residues could be detected in one sample. However, even in this setup, the newly established cMID assay demonstrates an easily applicable and powerful tool for on-site testing, which allows sensitive and reliable detection and quantification of penicillin G and kanamycin in WFM without laboratory-based cultivation equipment, absorbance measuring device or cost-intensive LC-MS/MS-based analysis methods.

## Figures and Tables

**Table 1:** Detected concentration of spiked penicillin G or kanamycin in WFM using cMID with calculated recovery rates (penicillin G: *n* = 4; kanamycin: *n* = 2).

Analyte	Concentration Spiked [ng·mL^−1^]	Concentration Detected [ng·mL^−1^]	Recovery Rate [%]
Penicillin G	2	1.83 ± 0.30	91.4 ± 14.9
	4	3.90 ± 0.56	97.4 ± 14.0
	10	10.03 ± 0.67	100.3 ± 6.7
	20	22.6 ± 2.61	112.9 ± 13.1
Kanamycin	1	0.88 ± 0.04	87.8 ± 4.3
	10	9.40 ± 0.01	94.1 ± 0.1
	50	26.26 ± 0.36	52.5 ± 0.7
